# Use of a Rapid HIV Home Test Prevents HIV Exposure in a High Risk Sample of Men Who Have Sex With Men

**DOI:** 10.1007/s10461-012-0274-2

**Published:** 2012-08-15

**Authors:** Alex Carballo-Diéguez, Timothy Frasca, Ivan Balan, Mobolaji Ibitoye, Curtis Dolezal

**Affiliations:** HIV Center for Clinical and Behavioral Studies at New York State Psychiatric Institute and Columbia University, 1051 Riverside Drive, Unit 15, New York, NY 10032 USA

**Keywords:** HIV, Rapid testing, Home testing, MSM, Harm reduction

## Abstract

The study assessed whether at-risk HIV-uninfected men who have sex with men (MSM) who never or rarely use condoms and have multiple partners would use a rapid, oral fluid, HIV home test (HT) to screen potential sexual partners. Participants received 16 HT kits, were monitored weekly for 3 months, and then interviewed in depth. Twenty-seven ethnically diverse MSM used HT kits before intercourse with approximately 100 partners in private and public spaces. Testing had high acceptability among ethnic minority participants. Ten tested individuals received HIV-antibody positive results. Seven were potential sexual partners, and three were acquaintances of the participants; six of the ten were unaware of their status. No sexual intercourse took place after positive tests. Very few problems occurred. Most participants strongly desired to continue using HT and to buy it freely. HT use results in detection of previously unknown infections. Making HT available within networks where high-risk sexual practices are common may be a cost-efficient and effective prevention method.

## Resumen

El estudio evaluó si los hombres que tienen sexo con hombres (HSH), que son VIH-negativos y tienen prácticas de riesgo –nunca o rara vez utilizan condones y tienen múltiples parejas– usarían una prueba rápida oral de VIH apta para el uso casero (HT por sus siglas en inglés), para determinar el estatus de VIH de sus parejas sexuales potenciales. Los participantes recibieron 16 equipos de HT, fueron monitoreados semanalmente durante tres meses y luego entrevistados en profundidad. Veintisiete HSH de diversas etnias, utilizaron los equipos de HT antes del coito con aproximadamente 100 parejas, tanto en lugares públicos como privados. El usar la prueba con parejas sexuales potenciales, tuvo una alta aceptabilidad entre los participantes de minorías étnicas. Diez individuos recibieron resultados positivos para VIH. Siete eran parejas potenciales y tres eran conocidos); de ellos, seis no sabían de su estatus seropositivo anteriormente. No hubo coito después de obtener resultados positivos. Hubo muy pocos problemas. La mayoría de los participantes expresaron fuertes deseos de seguir utilizando el HT y de poder comprarlo libremente. La utilización de HT da como resultado la detección de infecciones previamente desconocidas. El poner el HT a disposición de redes sociales donde las prácticas sexuales de alto riesgo son comunes, puede ser un método de prevención costo-eficiente además de efectivo.

## Introduction

Biomedical strategies for the prevention of HIV transmission recently have met with considerable success. Tenofovir gel applied vaginally was initially shown to decrease HIV transmission by 39 % among women who have sex with men [1] (although a later study [2] did not replicate the findings), and Truvada (emtricitabine and tenofovir disoproxilfumarate) pills ingested daily as pre-exposure prophylaxis (PrEP) showed a 44 % transmission reduction among men who have sex with men (MSM) [3]. Although both strategies showed only partial efficacy against HIV transmission, they were hailed as breakthroughs given that more than 30 years into the HIV epidemic many people at risk of HIV infection cannot or will not be abstinent or use condoms consistently. Alternatives are sorely needed. Consequently, discussions are underway for FDA licensing of Tenofovir gel and Truvada for PrEP purposes [4, 5]. Truvada as PrEP is also being piloted in community demonstration projects in San Francisco and Miami [6, 7].

A biomedical strategy that has received little attention despite its ready availability is the possible use of rapid HIV test kits at home (home testing or HT) to screen sexual partners. While advocates have touted the need for HT as a way to increase access to HIV testing, prompt earlier testing, and increase personal HIV status awareness and autonomy [8, 9], very few [10, 11] have recognized its potential for partner screening to reduce sexual risk. An HT kit that can deliver results almost immediately is not yet available for over-the-counter sale (OTC) in the United States, but this will soon change. The FDA recently approved for OTC sale the OraQuick In-Home HIV Test, an oral fluid test that requires no professional training for its administration or interpretation, can deliver results in 20 min, and has a sensitivity of 92 % and a specificity of 99.98 % [12]. An FDA advisory panel had previously unanimously recommended its OTC licensure [13, 14]. Once available, people may use HT to obtain information about the HIV status of a sexual partner prior to intercourse and to decide what protective strategy, if any, to use. A caveat is that OraQuick is an antibody test; therefore, an HIV-infected individual may appear uninfected until antibodies are generated. The window period of antibody tests lasts 25 days on average and in some cases as long as 8 weeks [15]. Thus, despite the high sensitivity and specificity of OraQuick, using it to screen sexual partners still would not completely eliminate risk. However, it would be another partially efficacious strategy for HIV-transmission prevention like the microbicide gels and PrEP currently being studied and piloted, as well as other existing HIV harm reduction approaches. For populations with high HIV prevalence such as MSM, and especially in urban areas where ethnic minority men have HIV-prevalence rates comparable to those of sub-Saharan Africa [16], HT could offer higher levels of protection than inconsistent condom use [17, 18]. Prior studies have shown that even among MSM who intentionally engage in unprotected anal sex when risk of HIV infection is present (“barebacking”) [19, 20] and who prioritize sexual pleasure and intimacy over protection against infection [21, 22] there is concern about and wish to avoid HIV infection [22, 23].

Our study was designed to test whether MSM who seldom or never use condoms and have sex in risky circumstances would use HT to screen sexual partners prior to intercourse. The Information, Motivation and Behavioral Skills (IMB) model [24] guided our inquiry into whether men with sufficient information on HT (including its limitations) would be motivated to use it to screen partners and what behavioral and negotiation skills they would employ to succeed at using HT for screening purposes. The results of the first stage of the study (hypothetical use), have already been published [11] (also see commentary [25]). Briefly, the men in the sample (*N* = 57) were able to understand the information we provided them on HT characteristics and limitations, specifically those referring to the window period and acute infection. Over 80 % of participants said they were motivated to use the kit to test sexual partners or themselves if the test became available OTC. Furthermore, 74 % of the participants in the first stage of the study demonstrated their skills by testing themselves unassisted in front of a research assistant in our offices and then correctly interpreting the test results.

We now present the results of the second (experiential) stage of the study. In this stage men were given HT kits to take home with the possibility of using them with their sexual partners over a 3-month period.

## Methods

Our study was conducted in New York City, USA, with approval from the New York State Psychiatric Institute Institutional Review Board. Recruitment took place in person and online at sites frequented by gay men with advertisement indicating that researchers were studying possible uses of a rapid HIV home test. Study candidates called the research office and responded to a few pre-screening questions. Those who qualified were invited to an in-person screening interview (Visit 1). After consent procedures, men were given a comprehensive description of rapid HIV HT, how it worked, and its window-period-related limitations. Subsequently, they took the first half of a 2-part, computer-assisted self-interview (CASI) that collected, *inter alia*, demographic information, HIV knowledge [26], sexual risk behavior in the prior 3 months, alcohol and substance use history, and prior history of STIs. Next, participants tested themselves with OraQuick following written instructions while monitored by a researcher. While waiting 20 min for the result of the test, participants completed the second part of the CASI. It included, among other sections, questions on whether the participant intended to use HT with partners when it became available OTC; his perceived capacity to discuss the use of HT with a partner and handle potentially positive results; his perceived ability to judge whether a partner could become violent and avoid or handle violent situations (adapted from [27]); and an 18-item questionnaire (true/false) specifically developed for this study on rapid HIV tests and their limitations, specifically the window period. Any incorrect responses to the last questionnaire generated feedback to the participant with the correct answer and its rationale; furthermore, the research assistant provided added clarification if necessary.

Negative HIV test results obtained with OraQuick at this visit and interpreted unaided by the participant were confirmed using a second rapid test (Clearview® Complete HIV 1/2) that is blood-based. The data collected during this screening process allowed us to determine participant eligibility.

Eligibility criteria included: man; 18 years of age or older; fluent in English or Spanish; HIV-negative; not in a monogamous relationship; engages in anal intercourse at least three times per month; never or seldom uses condoms (no condom use in last 10 occasions for those with 4 or less partners or in less than 80 % of occasions for those with more than 4 partners in the past year); aware that unprotected receptive anal intercourse (RAI) may lead to HIV transmission; understands the window period of OraQuick; reports likelihood of using HT to screen potential sexual partners; and feels he can avoid or handle potential violence resulting from proposing to use the test.

Study candidates who fulfilled eligibility criteria returned to the research offices on a subsequent day (Visit 2). After a new consent process, they enrolled in the 3-month study. They received a bag containing condoms, 16 HT kits, written instructions on HT kit use, a card with HIV- and violence-related resources available in the community, the study Website address, and a 24-h hotline number they could use for assistance from two senior clinical psychologists supervising the study. Participants were also trained on how to use an interactive voice response system (IVRS) to call at least once per week to report their sexual behavior and HT use. If no call was placed in a week, participants received an automatic reminder generated by the system. If they did not respond to the reminder, a staff member called them personally. Staff also called in response to any IVRS report of an HIV-positive result.

Visit 3 took place 3 months after Visit 2. Participants underwent an in-depth interview conducted by a clinical psychologist following an interview guide. The guide explored the constructs of the IMB model with specific attention to motivational factors that led participants to use (or not use) the test with different partners and the skills they employed to propose and use HT, interpret results, and handle partners’ reactions. Furthermore, in the course of this interview, a summary of the data collected through the IVRS was discussed and ambiguous issues clarified.

Participants received between $30 and $70 as compensation for their time at the different visits, plus a modest monetary incentive per call and a bonus if calls were received at least once a week, for a possible total of $190.

## Data Analyses

Quantitative CASI data were analyzed using SPSS [28] to calculate descriptive statistics.

In-depth interviews were recorded, transcribed, and checked for accuracy. Repeated reading of transcripts by a team of four researchers led to the identification of the main themes that constituted the basis for codebook development. Codes were defined with inclusion and exclusion criteria including examples. All transcripts were double-coded, and discrepancies discussed until reaching consensus. Codes were reviewed to identify modal responses, cases that contradicted the main trends, and quotes to be included in the text.

## Results

### Sample Description and Baseline Behavior

Approximately one out of eight potential study volunteers who contacted us passed the pre-screening criteria. Forty-four men initially qualified and were invited to attend the in-person screening Visit 1. Of these, 12 did not qualify for enrollment due to the following reasons: three tested HIV-positive; three did not feel capable of handling violent situations; three did not report qualifying risk behavior despite their initial pre-screening profile; one participant reported that he would not ask a partner to use HT under any circumstances; and one was unable to understand the window period of the test despite clarification from the research assistant. In addition, two participants were excluded when they provided contradictory or false data. (One participant was disqualified for two of the above reasons).

Of the 32 participants enrolled in the study, four did not complete all study procedures and a fifth case was discarded due to unreliable data. The men who completed the study did not differ from the excluded men in terms of their expressed likelihood to use the test on themselves; although they were marginally less likely to use HT with partners (*P* = .051), their answers were nevertheless within the likely-to-use range.

The final sample consists of 27 men. Table 1 presents their sociodemographic characteristics.

**Table 1:** Sociodemographic characteristics of participants (*N* = 27)

	Mean (SD) [range]
Sociodemographic characteristics	
Age	34.0 (11.4) [18–58]
Income (in thousands)	20,587 (22,863) [0–90,000]

	*n* (%)
Education	
High school graduate or less	9 (33 %)
Partial college	11 (41 %)
College graduate or more	7 (26 %)
Race/ethnicity	
White	11 (41 %)
Latino	4 (15 %)
Black	9 (33 %)
Asian/Pacific islander or mixed ethnicity	3 (11 %)

Table 2 shows that participants had engaged in significant sexual risk behavior in the prior 3 months as evidenced by their multiple partners and frequency of unprotected receptive and/or insertive anal intercourse. Almost all participants reported consuming alcohol in the prior 3 months, slightly over half used marijuana, and more than one-third used other drugs. Almost half of the participants had had an STI in the course of their lives, the most frequent being gonorrhea. Fifteen percent of the sample reported having had three or more STIs in their lives. By study design, all participants were HIV-uninfected. Eighty-eight percent of them reported having been tested for HIV within the past 2 years.

**Table 2:** Sexual risk behavior, substance use, and history of STIs (*N* = 27)

	Mean (SD) Mdn [range]
Sexual risk behavior in the prior 3 months	
Number of male partners	15.3 (17.8) 10 [3–90]
Unprotected receptive anal intercourse occasions (URAI)	10.8 (16.3) 4 [0–80]
Unprotected insertive anal intercourse occasions (UIAI)	9.1 (18.6) 2 [0–80]

	*n* (%)
Alcohol or drug use in the prior 3 months	
Alcohol	25 (93 %)
Marijuana	15 (56 %)
Poppers	10 (37 %)
Other	10 (37 %)
Lifetime history of STIs	
Ever had an STI	13 (48 %)
Gonorrhea	8 (30 %)
Chlamydia	4 (15 %)
Syphilis	4 (15 %)
Pubic lice	2 (7 %)
Other	6 (22 %)
Number of people reporting having had 1 STI in the past	6 (22 %)
Number of people reporting having had 2 STIs in the past	3 (11 %)
Number of people reporting having had 3 or more STIs in the past	4 (15 %)

### Utilization of HT Kits

According to IVRS and in-depth interviews, the participants had approximately 140 sexual partners during the 3 months of study. They proposed using the kit to about 124 men of whom 101 accepted and 23 refused. Ten tested individuals got HIV-antibody positive results. Seven were potential sexual partners and three were acquaintances of the participants; six of the ten were unaware of their status. Two participants each tested two partners who got positive results.

Below, we present excerpts from in-depth interviews that characterize in the participants’ words their experiences using HT. Each quote includes participant ID, age, and ethnic group in parentheses.

Participants liked having access to HT for use with partners and found it easy to use.

You just swipe it once on the top [gum], swipe it once on the bottom, and then put it in the test tube and stuff like that. It’s pretty easy. Simple. (#1014, 25 years old, AA)

Although about one-fifth of the partners refused to use the test and left the place of the encounter, most partners were receptive to using HT prior to sex.

People were a lot more willing to try the test than I was expecting,… and I had no problems, no hostility or anything toward me asking them to take the test. And it went perfectly fine. (#1035, 19, L)

Lack of partner resistance to taking the test was seen as a good sign. When partners resisted, participants often interpreted it as a warning not to have sex with that person.

And the ones that wasn’t with it, either I didn’t do nothing with them, or I used a—or I used a condom with them. Yeah, ‘cause I just didn’t trust it ‘cause I was thinking they was infected.” (#1014, 25, AA)

Most participants used HT at home, but 17 of them reported carrying the kits and using them at their partners’ homes or even in public places.

At this point, I start carrying some of them with me, the tests with me. So just in case anything happens, I have my little plastic baggie, open it up, do what we have to do. And we went to his place, and I said, you know, I really like you, this is really hot, and I think we can really have fun here. How would you like to test with me? (#1015, 33, W)

It was a little awkward to wait in the bathroom at the supermarket, waiting for the results. (#1035, 19, L)

Often, mutual testing took place.

I did it for him, and he did it for me. We opened the kits, you know, like put them side-by-side. And I swabbed him. He swabbed me. We put it in. And then we waited the 20 min, which seemed like a lifetime [laughs]. (#1017, 47, W)

Although waiting 20 min for the results provoked anxiety in four participants, the rest did not report such problems. They reported playing video games, watching TV, eating, drinking, “rolling weed,” smoking, or engaging in foreplay; some found the wait beneficial to ponder what to do next.

The 20-min window sort of gives you that extra 20 min to decide, “Okay, if this comes back negative, am I really ready to bareback?” (#1017, 47, W)

Substance use was frequent in this sample (only three participants reported no substance use); yet, in most cases it did not appear to hamper HT use.

If you would just meet somebody in a bar, having a few drinks, some of the inhibitions come down, the walls come down, and other topics of conversation are available to come above to the surface. So I think with the alcohol with the testing, I think it was just the way that I can incorporate the tests into the conversation or between our engagement as not a tool, but just as a preface to go on to do other things. (#1016, 43, W)

Two prospective partners knew they were infected with HIV and disclosed it to the participant when he proposed to use HT. In one case the partner stated that as he was planning on using condoms and his viral load was undetectable, there had been no need to disclose his status before. Conversely, six out of seven partners with reactive tests were unaware of their positive status and found it out using HT.

So we’re chatting at dinner, and it’s definitely leaning in the sexual area […] And it built up into let’s go to, you know, let’s go to your place […]. Matter of fact, when we get there, since I’m already part of this study, you know, about basically HIV awareness, and risk, and risk taking, why don’t I test in front of you, would you like to test with me? Cool, that would be great. Then we can have as much fun as we want. You know, and it went really, really well. And then he got the [positive] result [laughter]—He had no idea, I guess. […] He’s like, What do you mean? I’m pretty sure it didn’t give you a false positive, this is a, you know, this is pretty straightforward. Listen, I still like you, we can still fool around if you want to. You know, I don’t know what you’re going through at the moment. I know for me, when I found out about the Hep [Hepatitis C], I thought my world had come to an end. […] So if you want to hug, I’ll give you a hug, like, I’m here for you. […] And he got pretty upset, you know, it was hard to see that. I said listen, I’ll go with you, if you want to go to another clinic and get retested, if you want me to bring you down to, you know, one of the counseling centers, if you want to contact the people in my study, I can do that. He just said, you know, I’d really like to just kind of take some time alone. And I said, Are you going to be all right? You know, like, I worried he might do something crazy, you know, and I really didn’t want to see that happen. So, I gave, you know, I left him with my number, I said, If you need to call me, please do. You know, please leave all the sexual stuff aside, like, you’re another person, and I care, you know? We can always fool around later if you want to, but that doesn’t change how I feel about you and what I think of you. So, you know, nothing really happened after the testing, but as far as I know, he said he was going to go get services. [Afterwards] I left him a message and texted him, but I don’t want to be pushing it, obviously, because he’s going through a lot. (#1015, 33, W)

There were very few adverse experiences. Out of the approximately 124 occasions in which participants invited their partners to use HT, seven led to verbally aggressive situations (two participants reported two aggressive situations each); none of them resulted in violence towards the participant. In one case the sexual partner became angry with the participant when he proposed to use HT unexpectedly during the sexual event. In another case a partner who tested positive stomped on the test kit and started cursing; he did not attack the participant. The participant described this partner as a rough, belligerent type who could react negatively but with whom he decided to go anyhow and propose HT use. One participant whose partner reacted aggressively said that he (participant) always carries a weapon when he goes to someone’s home, thus indicating that he anticipates violence and gets involved in potentially violent situations. Of the four calls that were made to the hotline, all dealt with questions about interpretation of test results; none was due to a violent episode as a result of using HT.

No UAI occurred in any of the cases in which a participant found that a prospective partner was HIV-infected.

The people that I tested that actually came out positive, I didn’t proceed to have sex with them. […] I still hung out or whatever like that, but I didn’t wind up having sex though. It definitely put it in my mind that I shouldn’t have sex with the person. (#1030, 26, L)

Participants were three times more likely to report that using HT made them reduce their risk, be more cautious, practice safer sex, or think more about whom to have sex with than they were to report that use of HT made them more likely to have UAI.

Because I’ve been in the study […] I’m kind of ruined from having sex with people where I don’t know what their status is. But I’ll tell you this, I always ask now. That’s the closest I can come to knowing, and I want as much of that kind of feeling as I can get. And it always makes me wish I had the test to see if I was right. (#1021, 58, AA)

Participants felt they could trust the test, and although they thought the test was not for everyone, they hoped it would soon be available OTC

I wish there were tests for everything, for syphilis, for gonorrhea, for meningitis, for tuberculosis. That happened to me one time where somebody—oh, what a mistake. I met somebody in a bathhouse […] in St. Louis and bought him a ticket to come out and see me. And he never came. I got a phone call from his friend who said he died, passed away, that he had tuberculosis and AIDS, and he didn’t tell me either of these things. That’s why it’s good. Had that been the case that I would have mentioned to him on the phone, you know, do want to visit me in New York City? By the way, I have this test kit. Would you be willing to take this test? Then maybe he’d ‘fess up and say, Well, I better tell you the truth. I am positive. And then I would, Thank you very much. See you later. So I think this will definitely save lives, definitely. (#1020, 56, W)

## Discussion

Besides giving additional support to the findings of the first (hypothetical) stage of our study, the results reported here give proof of concept that HIV-uninfected MSM from diverse ethnic backgrounds who never or rarely use condoms and have intercourse with multiple partners understand the limitations of HT and are willing and able to use HT to screen partners. Most importantly, our results show that use of HT results in prevention of HIV exposure. The high yield of positive results (about 10 % of tested individuals were found to be infected) and the high proportion of partners (60 %) who were previously unaware of their infection show that HT may be a very effective and cost-efficient strategy for HIV detection. Moreover, availability of HT may result in more frequent testing among individuals with high-risk behavior, earlier detection of new infections, and distribution of test kits among network acquaintances presumably also engaging in high-risk sex. This is particularly promising in light of recent studies that have shown high rates of UAI among MSM who serosort based on the assumed serostatus of their partners (“seroguessing”) as well as infrequent and low HIV testing rates among serosorters [29–32].

Beyond actual use, the availability of HT and intention to use it may result in initiation of a discussion of HIV-related concerns and more honest disclosure of HIV-positive status from individuals aware of their infection. While prior studies have shown that MSM are less likely to disclose their HIV-positive status to casual or anonymous sexual partners than to main sexual partners [33–35], among our sample of MSM who had multiple casual partners, HT use led to several discussions on HIV prevention and prompted two partners to disclose their seropositivity. The method appeared to have ample acceptability not only among White MSM but also among ethnic minority MSM, a population hard hit by the epidemic for which many HIV-prevention approaches have failed and effective interventions are much needed [36].

The window period of the oral fluid test used in this study remains an issue—one upon which the biggest concerns about using HT to screen sexual partners will be raised. Yet, participants in our study understood and remembered the window period limitations. For instance, only one out of 44 individuals screened for the study was deemed ineligible to participate because he did not understand the concept of the window period. On the other hand, several participants referred to the window period while discussing their sexual behaviors during the in-depth interview at the end of the 3-month study. Furthermore, new tests are being developed that reduce the length of the window period. For example, Determine HIV-1/2 Ag/Ab Combo™, a fourth generation, rapid in vitro immunoassay qualitatively detects HIV p24 antigen as well as antibodies to HIV-1 and HIV-2 in serum, plasma, and whole blood. The p24 antigen is produced during the first few weeks of HIV infection and is detectable 7–9 days earlier than HIV antibodies. Test results can be read in 20 min [37]. Although this test is not yet available in the US, new rapid OTC tests undoubtedly will become available in the future with shorter window periods that could increase the prevention potential of HT.

Another potential barrier to the adoption of HT as a risk reduction strategy is the concern that it might lead users to take additional risk. Similar arguments have been raised in regards to other HIV risk reduction techniques including needle exchange programs—a successful strategy whose implementation was delayed for years despite ample research findings demonstrating its utility [38–40]. Just as the provision of clean needles to injection drug users was once feared to promote substance use, the use of HT to screen sexual partners is at this point a cutting-edge strategy that faces an uphill battle. While some may argue that people will “migrate” from condom use to the less reliable strategy of HT screening thereby increasing their HIV risk, it should be noted that our study was conducted with MSM who never or seldom use condoms. Therefore the adoption of HT among this population would not replace their sporadic condom use but instead provide them with an additional risk reduction option [17, 18].

Use of HT as a screening tool may result in public health savings much needed in times of budget constraints. For example, the current cost of OraQuick™ is less than $20 per kit, depending on the number of units purchased, and NGOs are working to reduce the price of rapid HIV testing kits [41]. By contrast, the estimated yearly cost of using Truvada™ as PrEP is $10,000 per person/year [42].

Generalization of our results should be made with caution. Our sample was small. Our eligibility criteria were very strict and resulted in a highly selective sample of MSM with high-risk sexual behavior and history of frequent STIs yet HIV-uninfected; they were recruited based on their stated intention to use HT and their professed self-confidence that they could handle potential violence. Therefore, they may not be representative of other MSM who engage in high-risk sexual behavior. Furthermore, the marginal difference between dropouts and completers in their intention to use HT with partners requires further study; it may indicate an effective opt-out of the technology by those who feel ill equipped to employ HT with partners.

Despite these limitations, our study highlights the important potential of use of HT as an HIV-prevention strategy for MSM who engage in high-risk behavior. If use of HT to screen sexual partners were to become widespread in high-risk sexual networks (e.g., barebackers), it could evolve into a community norm that could facilitate both discussion and use of the test. Guidelines on how to discuss HT with potential sexual partners (e.g., do it before the actual sexual encounter, suggest mutual testing, discuss with partner the resources available if someone tests positive before testing) may decrease the chances of untoward events. Furthermore, since the tactic is peer driven, it may empower individuals to take control of their behavior, develop a non-condom-based approach for communal, shared responsibility to prevent HIV transmission, and ultimately transform serosorting from a guessing game into a strategy based on objective evidence.
